# Placenta Accreta in a Woman with* Escherichia coli* Chorioamnionitis with Intact Membranes

**DOI:** 10.1155/2015/121864

**Published:** 2015-12-27

**Authors:** Emma M. Montelongo, Nathan R. Blue, Richard H. Lee

**Affiliations:** Department of Obstetrics and Gynecology, LAC+USC Medical Center, University of Southern California, 1200 N. State Street, IPT C3F107, Los Angeles, CA 90033, USA

## Abstract

*Background*.* Escherichia coli* (*E. coli*) associated intrauterine infections with intact membranes are extremely rare.* Case*. A 30-year-old multiparous female presented at 26 weeks' gestation with clinical signs of chorioamnionitis but physical examination suggested intact membranes. Her dietary history was concerned with Listeriosis. An amniocentesis was performed. Shortly thereafter, the mother developed septic shock and an urgent Cesarean delivery was performed. The patient required a peripartum hysterectomy for placenta accreta. Amniotic fluid cultures grew* E. coli*.

## 1. Introduction

Chorioamnionitis can be caused by a variety of bacterial species and few viruses, and it is rarely associated with intact membranes [1]. We report a case of chorioamnionitis due to* E. coli* in a woman with intact membranes and who also required a peripartum hysterectomy for placenta accreta.

## 2. Case

A 30-year-old gravida 4 para 3 presented to our hospital at 26 0/7 weeks' gestation with a 10-hour history of lower abdominal pain, vaginal discharge, dysuria, and complaints of subjective fever. She described the vaginal discharge as mucoid and blood-tinged. She denied any history of invasive procedures (i.e., amniocentesis) or leakage of fluid from her vagina prior to having the vaginal discharge. Her obstetrical history was significant for two prior term Cesarean deliveries. Upon presentation, she was afebrile and normotensive with a pulse of 106 beats per minute (bpm), no uterine contractions, and a nonreactive fetal heart tracing. Her maximum temperature was 100.0°F. Her abdominal exam revealed generalized tenderness. Speculum examination showed copious yellow mucopurulent discharge in the vaginal vault and the cervical os was closed. There was no pooling of amniotic fluid and testing for amniotic fluid ferning was negative. Nitrazine testing was mildly positive but was attributed to the mucopurulent discharge. Her white blood cell count was elevated at 17.9 [10^3^/*μ*L]. Ultrasound revealed a singleton fetus in breech presentation with an amniotic fluid index of 18.6 cm. The placenta was posterior fundal with no placenta previa. The placenta did demonstrate placental lacunae.

When interviewed, the patient acknowledged having consumed “queso fresco” throughout her pregnancy. Queso fresco is a Mexican cheese often made with raw, unpasteurized cow milk and is associated with Listeriosis when consumed in pregnancy. Because of this concern, an amniocentesis was performed with the plan to provide* Listeria* specific antimicrobial treatment if confirmed. When the amniocentesis was performed, it yielded yellow-opaque colored fluid, which was sent for Gram stain, culture, and glucose concentration and white blood cell count. Following the procedure, treatment with intravenous ampicillin and gentamicin was initiated. A few minutes later, the patient became tachycardic and tachypnic with rigors and chills, while complaining of extreme thirst. Fetal tachycardia was also observed. Given the concern for maternal sepsis due to chorioamnionitis, a Cesarean delivery was performed. A viable female infant was delivered weighing 790 grams with APGAR scores of 5 at 1 minute and 6 at 5 minutes. The placenta did not deliver with external uterine massage and was therefore removed manually. The placenta was removed with difficulty and extracted in segments. The placental bed was inspected and appeared hemostatic. The uterine incision was closed. Subsequently, the patient developed uterine atony which was treated with uterine massage and uterotonics. After closure of the laparotomy, the patient continued to have uterine atony. Bimanual compression and additional uterotonics were administered, and an intrauterine BAKRI balloon was placed. Her vital signs were stable, and she was extubated and transferred to the surgical intensive care unit. The total estimated blood loss was 1500 cc and her postoperative hemoglobin decreased from 9.6 [g/dL] to 7.5 [g/dL].

Despite these measures, the patient's uterus had intermittent atony. Her vital signs decompensated with a heart rate of 140 bpm and blood pressure of approximately 60/40 mmHg. Because of the lack of response to uterotonics and uterine compression, a peripartum hysterectomy was performed. The total estimated blood loss was 5000 cc, and the patient was transfused eleven units of packed red blood cells, three units of platelets, ten units of cryoprecipitate, and eight units of FFP. The intraoperative hemoglobin was 3.8 [g/dL] and postoperatively it was 13.3 [g/dL].

The amniotic fluid studies resulted in 4+ Gram-negative rods, which were later confirmed to be* Escherichia coli* by culture. Urine culture also grew* E. coli* but blood cultures resulted negative. Pathologic examination of the uterus identified placenta accreta. Examination of the umbilical cord and placenta revealed acute funisitis, meconium staining, and chorioamnionitis.

The patient continued to receive intravenous ampicillin and gentamicin for twenty-four hours following the operation. Her hospital course was complicated by an ileus, an acute rise in bilirubin that was attributed to sepsis, and a urinary tract infection that was treated with ceftriaxone. She was discharged on postoperative day ten. Immediately after birth, the neonate was found to have an elevated white blood cell count of 50 [10^3^/*μ*L] and was treated for presumed sepsis with intravenous ampicillin and gentamycin for fourteen days. She remained hospitalized for approximately three months in the neonatal intensive care requiring two months of intubation, closure of a patent ductus arteriosus, and total parenteral nutrition. At one year, she has had multiple hospitalizations for chronic lung disease and has been observed to have some developmental delays.

## 3. Discussion

Chorioamnionitis most commonly results from migration of cervicovaginal flora through the cervical canal to cause intra-amniotic infection, though transplacental infection can also occur through hematogenous spread or from direct inoculation during an invasive procedure. In this case, the presence of intact membranes initially lowered the concern for an ascending intra-amniotic infection from ruptured membranes.

There are numerous reports of intrauterine infection with intact membranes caused by a variety of agents, including* Listeria monocytogenes*,* Candida*,* Capnocytophaga sputigena*,* Fusobacterium nucleatum*,* Staphylococcus aureus*,* Streptococcus agalactiae*,* Eikenella corrodens*,* Streptococcus viridans*,* Haemophilus influenzae*, and some species of* Ureaplasma*. Transplacental infection with* Listeria* has been previously associated with unpasteurized “queso fresco” [2]. A comprehensive EMBASE and PubMed search using the search terms “*chorioamnionitis*,* Escherichia coli*,* intact*,* membranes, pregnancy*” yielded only one case report that* E. coli* related chorioamnionitis in a patient with intact membranes. In contrast to our case, the report described a patient who underwent placement of two cerclages. The authors report that the resulting infection was likely secondary to hematogenous spread from repeated instrumentation with the suture serving as a nidus for infection [3]. Besides this single report, it has been widely observed that intrauterine* E. coli* infection portends a poor prognosis to the fetus and has been associated with midtrimester fetal demise [4–7].

While there has been an observed association between placental lacunae and pathologic conditions such as placenta previa and accreta [8, 9], several large-scale studies have failed to demonstrate a significant association between this ultrasound finding and any adverse pregnancy outcome [10, 11]. The lack of consensus on the topic suggests that lacunae observed by ultrasound should not be interpreted as a diagnostic finding. What is unusual about this case is the location of the placental location and the accreta. Although the patient had a history of two previous Caesarian sections, the placenta was located on the posterior-fundal uterine wall and away from the anterior lower uterine segment, the area where both prior uterine incisions would be located. Given the unlikely event of de novo abnormal placentation remote from the uterine scar, our suspicion for placenta accreta prior to surgery was low.

The placenta accreta in this case is unlikely to have been caused by the chorioamnionitis. Distortion of the placental attachment to the uterus would have been expected to occur much earlier in the pregnancy. A recent population-based study of placenta accreta did not demonstrate any association between chorioamnionitis and abnormal placentation; however, the study was likely underpowered to detect a significant relationship as only three cases of chorioamnionitis were identified among 128 patients with placenta accreta (OR 0.88 CI (0.28–2.78)* P* value 0.83) [12]. In addition, there is some evidence to suggest that intra-amniotic inflammation may be associated with certain placental abnormalities, such as a placental edema and terminal villous immaturity [13]. Although the cause of her accreta is unknown, we speculate that the direct attachment of the placenta to the myometrium, rather than the decidua, might have facilitated the transmission of* E. coli* by the absence of the decidual layer that normally separates the myometrium from the placenta.

In conclusion,* E. coli* may now be included as a bacterium that can also cause chorioamnionitis in the setting of intact membranes. Although rare,* E. coli* associated chorioamnionitis can cause severe, life-threatening complications for both mother and fetus.
